# Intestinal Stem Cell Development in the Neonatal Gut: Pathways Regulating Development and Relevance to Necrotizing Enterocolitis

**DOI:** 10.3390/cells10020312

**Published:** 2021-02-03

**Authors:** Aparna Venkatraman, Wei Yu, Christopher Nitkin, Venkatesh Sampath

**Affiliations:** Division of Neonatology, Department of Pediatrics, Children’s Mercy Hospital, Kansas City, MO 64108, USA; avenkatraman@cmh.edu (A.V.); wyu@cmh.edu (W.Y.); crnitkin@cmh.edu (C.N.)

**Keywords:** intestinal stem cells, necrotizing enterocolits, morphogentic pathways, immune signaling, mesenchymal stem cell therapy, exosomes

## Abstract

The intestine is extremely dynamic and the epithelial cells that line the intestine get replaced every 3–5 days by highly proliferative intestinal stem cells (ISCs). The instructions for ISCs to self-renew or to differentiate come as cues from their surrounding microenvironment or their niche. A small number of evolutionarily conserved signaling pathways act as a critical regulator of the stem cells in the adult intestine, and these pathways are well characterized. However, the mechanisms, nutritional, and environmental signals that help establish the stem cell niche in the neonatal intestine are less studied. Deciphering the key signaling pathways that regulate the development and maintenance of the stem cells is particularly important to understanding how the intestine regenerates from necrotizing enterocolitis, a devastating disease in newborn infants characterized by inflammation, tissues necrosis, and stem cell injury. In this review, we piece together current knowledge on morphogenetic and immune pathways that regulate intestinal stem cell in neonates and highlight how the cross talk among these pathways affect tissue regeneration. We further discuss how these key pathways are perturbed in NEC and review the scientific knowledge relating to options for stem cell therapy in NEC gleaned from pre-clinical experimental models of NEC.

## 1. Introduction

The small intestinal tract is a tube-like structure that is critical for the absorption and digestion of nutrients as well as excretion of waste. Besides being a metabolic organ, numerous epithelial and non-epithelial cells play an important role in host immunity acting as a barrier to a multitude of commensal and pathogenic microbiota. The intestine is composed primarily of three tissue layers. The outer smooth muscle layer together with the enteric nervous system is responsible for peristalsis. The middle submucosal layer is the space between the outer muscle and the inner mucosal layer that is filled with stromal cells, nerve fibers, and various cells of the immune system. The inner mucosal layer consists of a single layer of epithelium with the lamina propria and basement membrane [1]. The single layer of intestinal epithelium along with mucin functions as a physical barrier to prevent the entry of luminal contents into the systemic circulation. This barrier can be disrupted with intestinal infections, bowel ischemia, or diseases involving severe inflammation such as necrotizing enterocolitis (NEC) and inflammatory bowel disease [2]. Where the damage is superficial, the epithelium is quickly repaired by the migration of adjacent cells in minutes to hours by a process called epithelial restitution [3]. However, in conditions where the lesions are more severe, intestinal stem cells (ISCs) in the crypt regenerate to replace the damaged epithelium over several days [4]. In this review, we discuss the signaling pathways that regulate intestine stem cell function in adults and in neonates. We discuss the pathogenesis of the major form of intestinal injury in the neonatal intestine, NEC, and review the literature with regard to application of stem cell therapy to treat NEC.

## 2. Intestinal Stem Cells

The intestinal epithelium is a continuously self-renewing tissue which serves a multitude of functions. Through its interaction with its surrounding mesenchyme, cells of the intestinal epithelium progress though repeated cycles to proliferate, terminally differentiate, and ultimately shed into the intestinal lumen. To fulfill this cellular demand, the small intestinal epithelium is organized into two spatially distinct units-the crypts of Lieberkühn which contain highly proliferative ISCs, and the long finger-like projections called villi which host terminally differentiated cells that are post-mitotic [5]. The intestinal stem cell (ISCs) resides at the base of crypt, and continuously divide to either self-renew or give rise to progenitors that differentiate to serve absorptive and secretory function. Absorptive progenitors differentiate into enterocytes that constitute most of the epithelium, and mediate nutrient absorption. Secretory progenitors differentiate into goblet cells, tuft cells, enteroendocrine cells, and Paneth cells. The secretory cells are involved in generation of mucin, hormones, and antibacterial agents [6]. All the differentiated cells except the Paneth cells move upwards along the crypt–villus axis and eventually die and slough off into the lumen. Paneth cells move downwards towards the crypt and are interspersed between the crypt based columnar cells (CBCs) or ISCs [7].

Physical positioning of the epithelial cells within the crypt have identified two subtypes of ISCs—the first subtype is the active intestinal stem cell called crypt base columnar cells (CBCs) which frequently divide and thereby contribute to majority of the tissue regeneration. These cells are wedged between Paneth cells and are identified by the marker leucine rich repeat containing G protein coupled receptor 5 (Lgr5). The second subtype of ISCs are the quiescent or reserve intestinal stem cells, which are slow dividing ISCs present in the +4 to +6 position from the base of the crypt. These quiescent ISCs, though resistant to stress, can be activated when the CBC function is compromised. Several proposed markers for +4 ISCs include but are not limited to are Bmi, mTert, Hopx, Dclk1, and *Lrig* [8]. Lineage tracing experiments have revealed that both populations of ISCs can self-renew and have the ability to give rise to all the lineages of the intestinal epithelium [4,9]. The ISC function is regulated by both extrinsic and intrinsic factors and have been described in detail elsewhere [10]. In brief, a gradient of morphogenic factors such as Wingless (Wnt) and Bone morphogenic protein (BMP) family members dictate ISC function to self-renew or to differentiate along the crypt–villus axis. In the crypts, Paneth cells and the surrounding mesenchyme/myo-fibroblasts constitute the niche environment. They generate several secretory and/or membrane-bound ligands from the Wnt, BMP, and Notch pathways that tightly regulate ISCs proliferation, maintenance, and differentiation [10,11]. Though the morphogenetic pathways are very well studied in the adult intestine, information in the developing neonatal intestine is limited. This review describes the progress so far made in identifying the developmental pathways that regulate stem cell ontogeny and tissue morphogenesis in the developing intestine and discuss how these pathways go awry in disease conditions like necrotizing enterocolitis affecting the neonates.

## 3. Neonatal Intestinal Ontogeny

In the early embryonic stage, the primitive gut epithelium develops as an undifferentiated pseudostratified layer from the endoderm with surrounding mesenchymal tissues. During later stages in fetal ontogeny, the epithelium differentiates into a monolayer of columnar epithelial cells forming villi. Unlike the adult mouse intestine, neonate mouse lack crypts at birth and the intestine matures postnatally [12]. Fully functional crypts along with Paneth cells appear two weeks after birth [13,14]. The proliferative epithelium is restricted to the intervillous region and penetrates the underlying mesenchyme to form crypts. The formation of the crypt–villus axis within intestinal tissue appears to be regulated at different levels: extrinsically by paracrine and endocrine signaling, and intrinsically by transcription factors and cofactors.

## 4. Paracrine Signaling Regulating Stem Cell Development

At the paracrine level, the cross talk between the surrounding mesenchyme and epithelium induce villus and crypt morphogenesis. The control of intestinal epithelial stem cell proliferation, differentiation and self-renewal are regulated by developmental pathways, which are evolutionarily conserved. These include the hedgehog (Hh), Wnt, BMP, and notch signaling pathways. These pathways overlap between organ morphogenesis and stem cell biology. Here, we describe the effect of evolutionarily conserved paracellular signaling on neonatal gut development.

### 4.1. Hedgehog(Hh) Signaling

Hedgehog signaling involves the binding of Hh ligands like sonic hedgehog (Shh), Indian hedgehog (Ihh), and Desert hedgehog (Dhh) to their transmembrane receptor Patched 1 & 2 (Ptch1 & 2) to release the Smoothened (SMO) signal transducer from its Patched-dependent suppression. SMO stabilizes Gli, the effector of Hh signaling and leads to Gli-dependent transcriptional activation of target genes [15]. During early development, the Hh ligands (Shh and Ihh) are expressed in the entire intestinal epithelium and become progressively restricted to the intervillus region, coinciding with villus morphogenesis. These ligands send signals to their corresponding receptors, Ptch1 and 2 expressed in the underlying mesenchyme, and subsequently to their effectors. Perturbation of the Hh signaling pathway by overexpression of a pan-hedgehog signaling inhibitor, Hedgehog-interacting (HhIP) impairs formation of villi, increases epithelial proliferation, increases Wnt activity, and decreases epithelial differentiation [16]. In addition, there is abnormal expansion and localization of the intestinal sub-epithelial myofibroblasts (ISEMF). Thus, proper patterning of ISEMF is essential for correct organization of the crypt–villus axis. Partial inhibition of Hh hedgehog signaling leads to abnormally branched villi with ectopic epithelial proliferation and ectopic activation of the Wnt pathway. This feedback mechanism of mesenchymal cells back to the epithelium is one of the finest examples of epithelial-mesenchymal interactions controlling intestinal organogenesis.

Another example of epithelial mesenchymal interaction leading to organogenesis is PDGF signaling. Expression of PDGF is predominantly in the intestinal epithelium, while its corresponding receptor PDGFR is in the mesenchyme. Loss of PDGFα ligand or its corresponding receptor affects villus morphogenesis and depletes surrounding PDGFR^+^ mesenchymal cells but does not affect epithelial proliferation or differentiation [17]. It is likely that Hh and PDGF signaling cooperate for mesenchymal remodeling and thereby regulate proper epithelial patterning. This relay of information from epithelium to mesenchyme and back to epithelium helps in the formation of the functional crypt–villus axis during postnatal development. Altogether, the hedgehog pathway in neonates play a key role in demarcating the villus from the crypt by promoting villus morphogenesis and restricting the proliferating compartment to the intervillus region.

### 4.2. BMP Signaling

As members of the TGF-β superfamily, BMPs traffic signaling between mesenchyme and epithelium. BMP signaling is initiated by binding of BMPs to their corresponding receptors—the most prevalent of which are type I (Ia or Ib) and type II. Binding of ligand to its corresponding receptor leads to the phosphorylation and translocation of the SMAD family of transcription factors, a downstream mediator for both TGFs and BMPs, into the nucleus [18]. These morphogens are important during development as well as in adults. In the intestine, while BMP ligands BMP2 and BMP4 are expressed in the surrounding mesenchyme, their corresponding receptor BMPR1a is expressed in intestinal epithelium. During early development, BMP4 expression is restricted to the intervillus region where nascent villi are formed [19]. Thus, the presence of phospho-Smad(p-Smad), an indicator of BMP activity, is seen mostly in the villus. Interestingly, the BMP antagonist noggin expression surrounds the base of the crypt. Taken together, the presence of BMP signaling inhibitor, Noggin, at the crypt base and BMP signaling activator/transducer, p-Smad, in the villi demonstrate a gradient of BMP signaling along the crypt–villus axis, with high BMP activity in the villus and low BMP activity near the crypt.

Germline mutations in *SMAD4* and *BMPR1a* are frequently found in individuals with familial Juvenile Polyposis. This condition is characterized by intestinal hamartomatous polyps comprised of both stromal and epithelial tissue [20,21]. Misexpression of the BMP inhibitor Noggin driven by the villin promoter in early stages of development results in enlarged and misshapen villi. Mice lacking *noggin* show an extensive loss of villi and ectopic crypt formation and hyperplasia of stroma similar to that of Juvenile Polyposis in humans [19,22]. Conversely, genetic ablation of the BMP receptor, or the overexpression of the antagonist *noggin* leads to development of a large number of crypt-like structures on the sides of the villi as well as where the crypts are localized [23]. Loss of BMP signaling in parallel activates Hh and PDGF signaling which could explain hyperplasia of the stroma. Altogether these studies suggest that, in neonates, BMP signaling plays a key role in villus morphogenesis, mesenchymal cell proliferation, and prevents ectopic crypt formation by cooperating with Hh and PDGF pathways.

### 4.3. Wnt Signaling

Wnt signaling is highly conserved across species and plays an important role in normal homeostasis and disease pathophysiology during development. Wnt ligands act as paracrine signaling molecules to initiate downstream signaling that include both canonical and non-canonical pathways. Canonical Wnt signaling is initiated by binding of Wnt ligands to their cognate membrane bound receptors the frizzled protein and co-receptor the LDL receptor-related proteins 5 and 6 (LRP5/6). Formation of this complex results in nuclear accumulation of β-catenin and subsequent activation of Transcription Factor 7 (TCF) target genes like cMyc. In non-canonical Wnt signaling, binding of Wnt ligands to the frizzled receptor stimulates downstream intracellular signaling independent of β-catenin, which influences various downstream pathways including the Ca^2+^-NFAT-IFN γ and the planar cell polarity (PCP) pathways [24,25,26].

Canonical Wnt signaling ligands (Wnt3,6 & 9b) as well as their corresponding receptors (Frizzled, 5–7 and Lrp5 and 6) are present only in the crypt epithelium, while members of non-canonical Wnt signaling are present in the Villus epithelium and the mesenchyme [27]. In the fetal stage and at birth, canonical Wnt signaling activity is evident only in the villus region. This is supported by several experiments that analyzed Wnt activity by measuring top-gal activity, nuclear localization of β-catenin, and β galactosidase staining. By postnatal day 2, Wnt signaling that was pervasively active across the entire villi during the fetal stage and covered the entire villi gets restricted to the intervillus region and ultimately to the crypt in adults. Homozygous deletion of TCF4, a key transcriptional effector of Wnt signaling in both neonates and adults, leads to a complete loss of crypts, and mice were left with non-dividing differentiated villus cells. This suggests that Wnt signaling is required for formation of crypts of Lieberkühn [28,29]. Similar results were obtained when other members of canonical Wnt signaling were perturbed. Ectopic expression of the Wnt ligand inhibitor Dickkopf1 (Dkk1), conditional deletion of β-catenin, and simultaneous inhibition of Lgr4 and Lgr5 all led to loss of crypts [30,31]. In addition, strong Wnt inhibition depleted the secretory cells but absorptive enterocytes remained unaffected. c-Myc, another transcription factor and downstream target of Wnt signaling follows the same expression pattern as that of nuclear β-catenin localization [32]. Similarly, conditional loss of c-myc, which is a direct target of the Wnt-β-catenin-TCF pathway, specifically from the intestinal epithelium of both adult and neonatal mice, was shown to inhibit crypt formation [33]. In contrast, transgenic expression of R-spondin 1 (R-Spo1), a strong Wnt activator, resulted in a massive hyperproliferation of intestinal crypts [34,35]. Taken together, these data indicate that Wnt signaling is essential for maintenance of crypt proliferation, intestinal stem cell function, and maintenance of secretory cells in neonates and adults. Loss of Wnt signaling parallels the loss of the crypt, and aberrant activation of Wnt signaling results in hyperproliferative crypts.

Wnt signaling also controls segregation of villus from crypt cells through Ephrin and Ephrin receptors. Akin to notch ligands, Ephrin ligands and Ephrin receptors are membrane bound and communicate with adjacent cells. These ligands and receptors repel each other and naturally create boundaries. In the neonatal mouse, ephrin B1 is expressed by the epithelial cells of the villi, and EphB2 and EphB3 by cells of the intervillus pockets. Genetic deletion of the EphB2 and EphB3 results in the abnormal presence of Paneth cells in the villus instead of being localized in the crypt. Post-mitotic cells present in the villus start appearing at the crypt base [36]. Thus, in addition to Wnt signaling regulating proliferation and differentiation, it also directs proper segregation of cell types in the intestine through ephrins.

### 4.4. Notch Signaling

Notch signaling is highly conserved and well recognized for its role in ISC proliferation and differentiation in the adult intestine [37]. Notch signals are transmitted between adjacent cells, such that Notch activity in one cell can induce distinct functions in a neighboring cell. This helps in establishing cell boundaries to pattern cellular differentiation and regulate stem cell function [38]. The Notch signaling pathway is highly conserved and has four well-known Notch receptors (Notch1–4) and 5 ligands (Jagged 1, 2 and Delta like ligand1, 2, 4) in mammals [39]. In the adult intestine, the role of Notch signaling in the regulation of intestinal epithelial cell proliferation and differentiation is well established. Modulation of Notch signals has profound effects on intestinal development. Inhibition of notch signaling components increases expression of atonal homolog 1 (ATOH1), a transcription factor, coupled with loss of crypt cell proliferation and secretory cell hyperplasia. A similar phenotype is observed when Atoh1 is transgenically overexpressed in the intestinal epithelium [40,41,42,43]. Conversely, constitutive activation of Notch signals in the neonatal intestine leads to an increase in the number of dividing cells and a dramatic impairment of differentiation of all intestinal cell types [44]. Ectopic Notch activation in the embryonic foregut results in reversible defects in villus morphogenesis and loss of the proliferative progenitor compartment. Unlike fetal and neonatal stages, ectopic Notch signaling in adult intestine leads to a bias against secretory fates [45].

Taken together, notch signaling appears to have variable effects at different stages of development in the intestine. While it predominantly affects proliferation in the fetal stage, in the neonates, it affects crypt proliferation and differentiation of all cellular lineages. In the adult intestine, however, it favors the absorptive fate and affects ISC proliferation. Furthermore, the effects of notch on proliferation requires cooperation with Wnt signaling molecules likeTCF4, whereas its influence on intestinal differentiation appears independent of Wnt [46].

## 5. Endocrine Signaling

Another layer of regulation for intestinal crypt formation is at the endocrine level. This is reflected in the steep increase in plasma thyroid hormone (T3) levels immediately after birth. In parallel, levels of the protein arginine methyltransferase 1 (PRMT1), a coactivator for the Thyroid hormone (TH)receptor, is upregulated strictly in the intervillus region. Lack of T3 or its receptor leads to a decrease in intestinal epithelial cells and abnormal intestinal morphology [47,48]. In addition to endocrine and paracrine control, recent studies have also shed light on the role of transcription factors in the intestinal morphogenesis, which form the focus of the next sections.

## 6. Transcription Factors

At the molecular level, the transcriptional repressor B lymphocyte-induced maturation protein 1 (Blimp1), a downstream target of the BMP/SMAD pathway, regulates intestinal maturation. Blimp1, which is strongly expressed in the entire intestinal epithelium throughout the fetal stage, becomes restricted to the intervillus region in neonates, and this phenomenon coincides with the rise in TH level. This suggests that Blimp1 expression is reciprocally regulated by Thyroid hormone activity.

Intestinal specific conditional deletion of Blimp1 led to enhanced formation of crypts, accelerated development of Paneth cells, and increased migration of enterocytes. Altogether, the conditional inactivation of Blimp1 compromises intestine function and survival [49,50]. This underscores the role of Blimp1 in intestinal maturation in neonates. Downregulation of blimp1 in the intervillus region where the stem cells are localized causes a concomitant increase in the expression of stem cell markers like Shh andPRMT1 [27,51]. In the adult intestine, the secretory lineage and crypt size is controlled by Wnt and notch signaling. However, it appears that, in neonates, BMP signaling predominates in this role, since Blimp1 acts downstream of BMP/SMAD signaling. It is also surprising that, while the loss of Blimp1 severely compromised intestinal function and mice survival in neonates, expression of members of Wnt and Notch family remained unaffected.

Sox9, a transcription factor and a downstream target of Wnt signaling, is expressed throughout the duodenal epithelium as early as embryonic day 13.5 (E13.5) and subsequently restricted to the crypt of the adult intestine. Sox9 represses Cdx2 and Muc2, two genes involved in epithelial differentiation, implicating it in intestinal stem cell differentiation. Conditional loss of Sox9 in the adult intestinal epithelium also led to increased proliferation and Paneth cell loss, [52,53]. However, the role of Sox9 in neonates remains unexplored.

Altogether, these studies illuminate the fact that a plethora of endocrine, paracrine, and transcription factors orchestrate intestinal morphogenesis from the neonatal stage to adult. The involvement of factors involved in the process during the neonatal period declines over time during maturation to adulthood, suggesting that adult stem cells have unique requirements of factors and cofactors for intestinal stem cell maintenance and function distinct from what roles that were predominant in neonates.

## 7. Necrotizing Enterocolitis Pathophysiology

Necrotizing enterocolitis (NEC) is the most common, life-threatening gastrointestinal emergency in the neonatal period. Mostly afflicting premature infants born at <36 weeks gestation, the incidence varies between 5 to 10%, with a mortality of 15–30% [54,55]. Clinically, NEC is characterized by abdominal distension, feed intolerance, bloody stools, thrombocytopenia, and escalating signs of cardiovascular shock in severe cases [54,56]. The classical abdominal radiograph findings of pneumatosis intestinalis (air within the intestinal wall), portal venous gas, or pneumoperitoneum is highly indicative of the diagnosis [56,57]. The major risk factors for NEC include prematurity, formula feeding, the combination of anemia and blood transfusions, hypoxic-ischemic events, and clinical chorioamnionitis [54,55]. Research in the last two decades has highlighted the role of development dysmaturity of the intestinal immune system, aberrant intestinal inflammation, gut microbiota, and genetics in the causation of NEC [58]. While the final common mechanistic is yet to be elucidated, consensus is emerging that NEC is a phenotype for deviant activation of intestinal Toll Like Receptor (TLR) signaling that results in a breakdown of the intestinal barrier, bacterial invasion, pathological intestinal inflammation, and necrosis, and, in severe cases, cardiopulmonary involvement and shock [54,55,58,59,60].

### 7.1. Dysmaturity of the Intestinal Immune System in Preterm Neonates

The premise for a dysmature intestinal immune response in NEC is founded on the fact that NEC is rare in full term neonates, and occurs in the setting of ischemia related to congenital heart disease or gastroschisis [60,61]. Data from mice suggest that innate immune signaling, especially TLR4 signaling is important for crypt development and intestinal epithelial cell (IEC) differentiation in the fetus [62]. After preterm birth, persistent IEC TLR4 signaling is potentially pathogenic, as gut bacteria can induce intestinal inflammation and necrosis through TLR4. In the term neonate, there is a rapid desensitization of IEC TLR signaling and acquirement of postnatal intestinal tolerance [63,64,65]. This is achieved by decreased expression of IRAK1, a key mediator of TLR signaling, increased expression of inhibitor of kappa B, an inhibitor of NFκB, and localization of TLR receptors including TLR5, to the basal from the luminal surface of IEC [63,64,65,66,67]. This precise temporal regulation of IEC TLR signaling during intestinal development highlights the dual role of TLRs in IEC, i.e., maintaining mucosal homeostasis and IEC function versus contributing to acute inflammation and necrosis (tolerance vs. intolerance). The concept that there is imbalance between pro-inflammatory and anti-inflammatory immune signaling in the preterm intestine is further demonstrated by studies showing that inhibitors of TLR signaling such as SIGIRR, A20, and TOLLIP show reduced expression in preterm intestine [68]. Interestingly, breast milk and probiotics, both of which protect against NEC, upregulate expression of genes that tamponade TLR4 signaling in the mouse intestine [69,70,71].

### 7.2. TLR4 Signaling and NEC Pathogenesis

**Figure 1:**
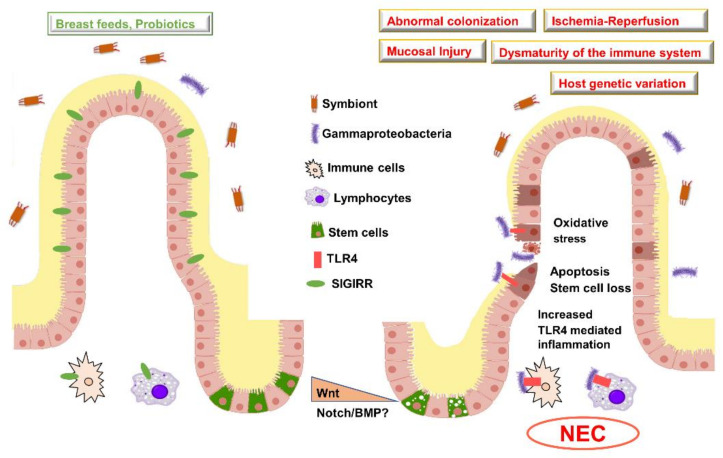
Risk factors and pathogenesis of NEC. Illustration of a healthy villus (**left**) versus villus injured in NEC (**right**). In preterm neonates who do not develop NEC, there is an intact mucin barrier, gut colonization with symbionts, less pro-inflammatory signaling, and increased expression in anti-inflammatory genes such as Single Immunoglobulin Interleukin-1 Related Receptor (SIGIRR). In infants who develop NEC, there is aberrant activation of Toll Like Receptor in association with increased *Gammaproteobacteria*, pro-inflammatory signaling, and bacterial translocation accompanied with reduced mucin barrier. Early studies suggest that, in NEC, Wnt signaling may be reduced accompanied with the loss of intestinal stem cells. Wnt, Wingless related integration site; BMP, Bone morphogenetic protein; TLR, Toll-like receptor; NEC, Necrotizing enterocolitis.

Human studies on intestinal tissue obtained from infants with NEC show increased expression of TLR4 and related downstream cytokines and chemokines, while inhibitors of TLR signaling such as SIGIRR are decreased [69,70,71,72]. Furthermore, microbiota studies show relative abundance of Gram-negative bacteria in preterm infants who develop NEC implying that aberrant activation of TLRs is central to pathogenesis of human NEC [73,74]. The definitive proof that TLR4 signaling is important for NEC pathogenesis was shown in mouse models of experimental NEC. C3HeJ mice that lack functional TLR4 signaling are protected against experimental NEC compared to TLR4 sufficient C3HeN strain mates [75]. Furthermore, IEC-specific deletion of TLR4 was protected against experimental NEC, demonstrating the critical role for IEC TLR4 signaling in initiating NEC pathogenesis [62]. Fawley et al. [76] showed that mice lacking SIGIRR, a negative regulator of TLR and Interleukin signaling, had more severe experimental NEC. This is particularly relevant to human NEC pathogenesis as the loss of function mutations in SIGIRR were enriched in preterm infants who developed NEC [77]. These data evince that TLR4 activation is required for NEC in humans, and in experimental NEC animal models (Figure 1).

### 7.3. Alternate Pathways Involved in NEC

While TLR4 activation is a major mechanism for NEC pathogenesis, investigators have identified other pathways that are relevant to NEC pathogenesis. Paneth cells lining the crypt of Lieberkuhn are key components of the innate immune system by secreting anti-microbial peptides [78,79]. Paneth cell ablation either through genetic targeting or chemical methods exaggerates NEC in the neonatal intestine [79]. Platelet activating factor (PAF) has been studied both in humans and mouse models, and contributes to intestinal inflammation and loss of barrier function seen in NEC [80,81]. In mice, inhibitors of PAF suppress inflammation and intestinal necrosis seen in experimental NEC [81,82]. Furthermore, in preterm infants with NEC, there is increased PAF expression in intestinal tissue as well as in the systemic circulation [83]. The interactions between TLR4 activation and PAF/Paneth signaling in NEC evolution remain yet to be elucidated.

### 7.4. Role of the Gut Microbiome in Programming NEC Vulnerability

The recent explosion in 16S rRNA sequencing and metagenomics-based studies have unearthed valuable information on neonatal gut microbial assembly and acquirement of a dysbiotic gut microbial signature that predisposes to NEC. While this is a vast area of research, a few points that are pertinent to this review are briefly discussed. Microbial assembly in the preterm infants is affected by several prenatal factors including maternal diet, presence of intraamniotic infection, and mode of delivery (vaginal vs. cesarean section) [84,85,86,87]. After birth, the major determinants of gut microbial assembly included type of feeding, i.e., formula feeding vs. breast milk, use of broad-spectrum antibiotics and the neonatal intensive care environment and practices [84,88,89,90]. Importantly, in comparison with term infants, the preterm infant has decreased diversity, with relative enrichment of *Enterobacteria*, and decreases in *Bacteroides* and *Bifidobacteria* [73,85,91,92]. In the preterm infant, infants who develop NEC tend to have increases in *Gammaproteobacteria*, a class of Gram-negative bacteria that encompasses many known pathogens implicated in NEC and sepsis in preterm infants including *Enterobacter*, *Klebsiella*, *E. coli*, and *Psuedomonas* [73,88,93]. While this is the most consistent pattern of signature associated with NEC, others have noted decreased bacterial diversity, and both a delay in acquirement and decreased abundance of *Bifidobacteria* and *Bacteriodes* [73,94]. The current concept of NEC pathogenesis revolves around luminal *Gammaproteobacteria* penetrating the immature intestinal barrier in preterm neonates and triggering exaggerated TLR signaling, which results in the intestinal inflammation and necrosis (Figure 1).

### 7.5. Genetic Susceptibility to NEC in Preterm Neonates

Over the last decade, the contribution of genetic risk factors to NEC susceptibility has been investigated by several investigators. Genetic risk factors can seek to explain the inter-individual differences in NEC vulnerability noted in premature infants who have similar clinical risk factors. Genetic variants in innate immune genes, vascular tone/growth regulating genes, anti-oxidant response genes, and cytokine/chemokine genes have been major areas of research [95]. Not surprisingly, one of the strongest genetic signatures to emerge is around TLR and innate immune genes. The presence of one or more loss of function NOD2 variants appear to predispose to NEC in one of the largest studies undertaken in premature infants [96,97]. In addition, using a sequencing based approach, rare and novel loss of function variants in single immunoglobulin interleukin 1 related receptor (SIGIRR) that exaggerate TLR4 signaling were enriched in preterm infants who developed NEC in a pilot study [77]. Interestingly, both NOD2 and SIGIRR are inhibitors of TLR signaling in the neonatal intestine [93,98]. Genetic variants in the VEGF and other vascular genes might also be potential loci for NEC vulnerability [99]. Genome-wide transcriptome studies in intestinal tissue obtained from preterm infants with and without NEC also identify innate immune pathways as a key target in NEC [100].

## 8. Morphogenetic Pathways and NEC

Under normal homeostatic conditions, the epithelial barrier is maintained by an intact crypt–villus axis. Transit amplifying cells arise from the ISC present in the crypt, migrating and differentiating along the villus. Finally, enterocytes undergo apoptosis and are shed into the lumen. This entire process takes 3–5 days. However, in conditions like NEC, the gut barrier function is impaired, and it is now increasingly recognized that the Toll like receptor, TLR4, plays a key role in the maintenance of epithelial barrier integrity. TLR4 is predominantly expressed in the crypt and was found to be colocalized with LGR5^+^ ISCs [101]. In both humans with the disease as well as in mouse models of NEC, there is an increased expression of epithelial TLR4. In mouse models of NEC, activation of TLR4 signaling increases enterocyte apoptosis and impairs epithelial restitution by affecting their migration. Activation of TLR4 signaling also led to reduction in epithelial proliferation suggesting a defect at the stem cell level [102,103]. Mechanistically, TLR4 activation increased intestinal apoptosis via the p53-upregulated modulator of apoptosis (PUMA). Mice lacking PUMA were not affected, despite having TLR4 activation. TLR-4 activation can also inhibit signaling by β-catenin, a member of the canonical Wnt signaling pathway, through upregulation of inhibitory kinase GSK3β. In contrast, mutations in TLR4 or adenoviral-mediated inhibition of TLR4 signaling in neonatal, but not adult, mice restored β-catenin/Wnt signaling and reduced disease severity, which was attributed to increased enterocyte proliferation [104]. Transcriptional profiling of ileal samples from an experimental model of NEC in mice demonstrate a decrease in the Wnt ligand, specifically Wnt 7b. In addition, accumulation of nuclear β-catenin in the intestinal epithelium was noted in both mouse models and patient samples. Altogether, it is evident that significant perturbation of Wnt signaling occurs in NEC [105].

In patients with NEC as well as in the mouse model of NEC, a significant reduction in the formation of the goblet cell is observed [62]. A lack of goblet cells affect mucin production and thereby compromise epithelial barrier integrity. This can be influenced by the notch signaling pathway, which plays a critical role in cell fate decision. Activation of Notch suppresses goblet cell formation via repressing ATOH1/Math1. In the mouse model of NEC, enterocyte-specific lack of TLR4 significantly increased goblet cell formation, an effect mediated through repression of notch signaling. This signaling was found to be via a Myd88-independent pathway. In addition, inhibition of notch signaling using the gamma secretase inhibitor DBZ in an animal model of NEC increased goblet cell formation and reduced severity of NEC, underscoring the importance of notch signaling in NEC pathophysiology [62].

Taken together, these studies demonstrate that immune signaling cooperates with morphogenetic pathways like Wnt and notch and contribute towards the development of NEC. These findings also illuminate differences in the role of TLR4 signaling in the neonate versus adult intestine (Figure 1). TLR4 is pro-apoptotic and inhibits stem cell proliferation in neonates, whereas, in adults, it promotes epithelial proliferation. Altogether, an increase in TLR dependent signals in neonates is central to NEC pathogenesis, while the absence of TLR signaling impairs epithelial regenerative responses [106] and affects the outcome of diseases like colitis.

## 9. Stem Cell Therapy for NEC—Sources and Mechanism of Action

Mesenchymal stem cells (MSCs) were first noted for their ability to regenerate infarcted myocardium [107], and are defined by their ability to differentiate into chondrocytes, osteocytes, and adipocytes [108]. Since their discovery, they have been identified in the bone marrow, adipose tissue, umbilical cord (Wharton’s Jelly), umbilical cord blood, placenta, and amniotic fluid [109]. Bone marrow-derived MSCs (BM-MSCs) were the first described MSC and are well-studied [110], but those obtained from the umbilical cord tissue (UC-MSCs) or umbilical cord blood (UCB-MSCs) may be a superior source [111]. UC/UCB-MSCs can be obtained in large quantities without invasive procedures, and appear to have greater immunomodulatory potential than BM-MSCs [112].

Contrary to the initial belief, MSCs do not directly become new tissue, but instead secrete growth factors and cytokines, as well as packaged exosomes and microvesicles containing protein, lipid, and nucleic acids to mediate beneficial effects [113]. Paracrine factors are key drivers of MSC efficacy; indeed, cell-free preparations are typically as effective as MSCs themselves [114]. Both MSCs and MSC-derived products act via anti-inflammatory, pro-angiogenic, anti-fibrotic, and anti-oxidant [115] mechanisms, mediated by factors such as IL-1β [116], IL-6 [116,117], TNFα [116,117,118], IFNγ [118], vascular endothelial growth factor [119], and TGF-β1 [120]. Because of their anti-inflammatory, pro-angiogenic, and immunomodulatory roles, they have been studied in adult stroke and myocardial ischemia, as well as sepsis and septic shock [121]. Thus, MSC therapy for NEC is attractive, as the pathophysiology of NEC includes bacterial infection, inflammation, and ischemia. There is also a relatively long history of MSC therapy for intestinal inflammatory disease, with moderate-to-severe Crohn’s Disease being one of the most advanced indications to date [122].

## 10. Stem Cell Therapy in Preclinical Models

MSCs for necrotizing enterocolitis (NEC) have been evaluated in animal models for the last decade, with multiple studies and a recent review [123]. Besner and colleagues have demonstrated the utility of amniotic fluid-derived MSC, BM-MSC, and neural stem cells in reducing the incidence of NEC in rats [124,125] with concomitant decreases in intestinal permeability [126]. Exosomes from these cell types were similarly effective [114,127], making cell-free therapy a realistic possibility. However, the source of exosomes is important as differential effects on disease progression have been noted based on the source. For example, exosomes released from intestinal epithelial cells or immune cells help in progression of NEC while exosomes derived from MSCs or variable milk source ameliorate the severity of NEC [128]. Milk-derived exosomes reduce oxidative stress, decrease cell death, restore goblet cells, muc2 production, and ER function in normal as well as in experimental NEC [129,130,131,132,133]. MSC-derived exosomes ameliorate the severity of experimental NEC and preserve gut barrier function in vivo [134]. At present, the mechanisms behind these diverging outcomes dependent on exosome source is not clear. Thus, additional studies are warranted to identify the downstream targets activated by different sources of exosomes.

Synergistic therapy with other novel interventions, such as exogenous heparin-binding EGF-like growth factor [135] and extracellular vesicles (EVs), also make such therapy more attractive. Extracellular vesicles (EVs) act as mediators of intercellular signaling via the delivery of effector molecules. Small intestinal fibroblasts derived EVs carry EGF family members that are growth factors required for ISC regulation. MSCs or stem cells derived EVs attenuate NEC intestinal injury by endogenous modulation the Wnt/β-catenin pathway, which may explain their prolonged duration of action despite their rapid clearance [66]. Stem cells derived EVs also stimulate intestinal recovery from NEC by increasing cellular proliferation, reducing inflammation and ultimately regenerating a normal intestinal epithelium [136]. Since providing niche factors is critical in homeostasis as well as during regeneration, EVs can serve as novel tools for transporting stem cell factors in disease conditions like NEC [137]. This may also provide an underpinning to the protective effect of breast milk, where enteral breast milk exosomes preserve expression of GRP94, which is upstream of Wnt [138]. Intriguingly, enteral administration of MSC also appears to be effective, despite the administered product being subject to the digestive process, and enteral administration of MSC-derived exosomes may also provide an effective and alternative option for preventing NEC [139]. Modulation of intestinal microbiota dysbiosis, a key feature of NEC pathogenesis [88], may be an additional mechanism by which MSCs prevent NEC. Adipose tissue-derived MSCs administered to rats in the cecal ligation and puncture model of sepsis reduced the proportion of harmful bacteria and increased the proportion of beneficial bacteria [140].

Finally, although there are no current clinical trials of MSCs or MSC-derived exosomes for NEC, an interesting case report for MSC therapy in NEC was published [141]. A 22-day old infant born at 37 weeks gestational age developed supraventricular tachycardia with subsequent NEC involving nearly his entire intestine. Sixty centimeters of bowel were removed, so the authors recommended a UC-MSC infusion, after which intestinal blood flow increased. Although this n-of-1 experiment needs to be pursued in larger trials, this and other trials for other preterm diseases have not reported major adverse effects. Although there are significant barriers to enrolling neonates in MSC trials [142], demonstrated safety might facilitate translation of these preclinical findings into clinically apparent benefits. It is also possible that preterm infants enrolled in MSC trials for other indications (e.g., bronchopulmonary dysplasia) may be incidentally noted to experience lower incidence of NEC.

## 11. Conclusions

The conserved morphogenetic pathways that regulate the maintenance of the stem cell in the adult intestine, and aid in tissue regeneration during disease are relatively well characterized compared to the neonatal intestine. Early studies suggest that the intestinal microbiota, IEC innate immune signaling, and metabolites in the neonatal intestine shape the development of the intestinal stem cell. Morphogenetic pathways including Wnt, BMP, Hh, and Notch may have distinct roles in the neonatal intestine, different from that described in the adult intestine. Understanding how the stem cell is generated, maintained, and disrupted in diseases such as NEC in the neonatal intestine will enable translating the power of stem cell therapy to improve outcomes for neonatal diseases.
